# Disparities in fertility knowledge among women from low and high resource settings presenting for fertility care in two United States metropolitan centers

**DOI:** 10.1186/s40738-020-00084-1

**Published:** 2020-08-15

**Authors:** Jacquelyn R. Hoffman, Meaghan A. Delaney, Cecilia T. Valdes, Diana Herrera, Samuel L. Washington, Lusine Aghajanova, James F. Smith, Christopher N. Herndon

**Affiliations:** 1 University of Arizona College of Medicine-Tucson, 1501 N Campbell Avenue, Tucson, AZ 85724 USA; 2 Department of Obstetrics & Gynecology, Baystate Medical Center, 759 Chestnut Street, Office S1661, Springfield, MA 01199 USA; 3 Department of Obstetrics & Gynecology, One Baylor Plaza, BCM 610, Houston, TX 77030 USA; 4 Department of Obstetrics & Gynecology, The Houston Methodist Hospital, 1401 St. Joseph Pkwy. Susan K. Strake Building, 1st Floor SKS1106A, Houston, TX 77002 USA; 5 Department of Urology, University of California, 400 Parnassus, Box 0738, San Francisco, CA 94143 USA; 6 Division of Reproductive Endocrinology & Infertility Services, Department of Obstetrics & Gynecology, Stanford University School of Medicine, 1195 West Fremont Avenue, MC 7717, Sunnyvale, CA 94087 USA; 7 UCSF Philip R. Lee Institute for Health Policy Studies, 3333 California St, San Francisco, CA 94118 USA; 8 Division of Reproductive Endocrinology & Infertility, Department of Obstetrics & Gynecology, University of Washington School of Medicine, 1959 NE Pacific St, Box 356460, Seattle, WA 98195-6460 USA

**Keywords:** Reproductive health knowledge, Socioeconomic status, Access to care, Disparities, Infertility

## Abstract

**Background:**

Few studies have examined health literacy and fertility knowledge among women from low income, socio-culturally diverse communities presenting for fertility care in the United States. Our study sought to examine demographic predictors of fertility-related knowledge among infertile women from low and high-resource communities in two major metropolitan centers in the United States.

**Methods:**

Fertility Knowledge Assessments were administered to women presenting for fertility care at county medical centers serving low-resource, largely immigrant patients and to women from largely affluent populations presenting to comprehensive fertility centers in two cities. The influence of demographic predictors on fertility knowledge was examined through regression analysis.

**Results:**

A total of 143 women were included in our analysis. In the county hospital/low resource clinic (LR, *n* = 70), the mean age was 32.8 ± 6.1 years vs 35.0 ± 5.0 years in the fee-for-service/high resource clinic (HR, *n* = 73). Among the LR patients, 74% were immigrants, 71% had an annual income <$25,000 and 52% had completed high school. Among HR patients, 36% were immigrants, 60% had an annual income >$100,000, and 95% had some college or above. On average, women from HR settings scored 3.0 points higher on the Fertility Knowledge Assessment than their LR counterparts (*p* < 0.001). Upon multivariate analysis, education level remained the sole independent factor associated with fertility knowledge assessment score (*p* < 0.001). Stratifying by resource level revealed that income was highly associated with fertility knowledge (*p* < 0.01) among high resource individuals even when adjusting for education level.

**Conclusions:**

Women from low resource, largely immigrant communities, seeking fertility care have greater disparities in fertility knowledge and lower health literacy compared to women from high resource clinical settings. Further studies are needed to understand these barriers and to develop targeted inventions to lower disparities and improve care for these vulnerable populations.

## Background

Low health literacy has been extensively correlated with poor health outcomes and reduced use of health resources across a range of conditions including cancer, diabetes, and asthma [1]. As an example, reduced health literacy is associated with lower cervical cancer screening rates and a higher incidence of cervical cancer, particularly in low-resource communities in the United States that carry the highest burden for risk [2]. Low health literacy has also been associated with poor contraceptive knowledge within resource-limited, minority populations [3–5].

Infertility is a disease of reproductive health that exerts a profound impact on an estimated 80 million people worldwide. The World Health Organization ranks infertility as the 5th highest generator of disability among the global population of all people under 60 years of age [6]. An increasing number of studies have examined health literacy in the domain of fertility knowledge [7–16].

In a large sample of infertile women of various ages and educational backgrounds across Canada, Daniluk et al. found that the majority of women lacked significant knowledge related to age-related fertility and ART options, treatment limitations, and their associated costs [9]. Vassard et al., in their comparative survey on fertility awareness among men and women in the UK and Denmark, similarly found that participants were unaware of age-associated impact on fertility [7]. In a recent study, Mu et al., using an online fertility knowledge assessment, found that young women had general knowledge about female fertility and conception but lacked accurate understanding of critical details such as the window of fertility in the menstrual cycle [12].

A systematic review of the available evidence on fertility awareness worldwide, including 71 studies published between 1994 and 2017 among 26 countries, found that overall fertility awareness is low to moderate among reproductive-aged women and that higher education correlated with greater fertility awareness [11]. The majority of the aforementioned studies primarily assessed fertility knowledge among women from private infertility clinics, university settings, those on social media sites, or medically trained resident-physicians. As such, these methods most likely did not include assessment of knowledge of women from lower resource, socio-culturally diverse backgrounds with lower access to resources. In these populations, health literacy is anticipated to be comparatively the lowest and its impact on health and accessing medical care the highest [9].

No study to our knowledge has quantitatively and comparatively examined fertility knowledge among infertile women within the United States across socio-economic background strata, and specifically within largely immigrant communities [17–19].

We sought to test the hypothesis that socio-economic and demographic factors (e.g. income level, region of origin) are associated with respondents’ fertility knowledge level. This, in turn, may serve as an indirect pathway to measure barriers to access to fertility care.

## Methods

### Description of study sites

We designed a multi-institutional cross-sectional study, using paired clinical sites at the University of California, San Francisco School of Medicine (UCSF), San Francisco, CA and Baylor College of Medicine (BCM), Houston, TX. All study procedures were reviewed and approved by the Institutional Review Board at each university.

The low-resource (LR) locations comprised of infertility clinics operating within the infrastructure of ambulatory women’s health clinics at university-affiliated urban public teaching community hospitals: San Francisco General Hospital and Ben Taub Hospital in Houston. These clinics operate weekly and provide free and low cost medical care to a largely immigrant, culturally diverse, and indigent population. Medical students and OB/GYN residents are supervised by a board-certified Reproductive Endocrinology and Infertility (REI) specialist or a senior REI fellow.

The high-resource (HR), fee-for-service sites included the UCSF Center for Reproductive Health (CHR) and the Texas Children’s Family Fertility Center at Texas Children’s Hospital Pavilion for Women. These comprehensive tertiary fertility care centers are largely self-pay and offer services including intrauterine insemination (IUI), in-vitro fertilization (IVF), fertility preservation programs, and ovum donation programs.

### Description of study participants

Infertile women desiring conception presenting for initial care at study sites were eligible to participate. Infertility was defined as the inability to conceive after 12 months of regular unprotected intercourse for women < 35 years old or 6 months for women ≥35 years old. Women presenting for infertility consultation were potential candidates for enrollment. Pregnant or women actively undergoing infertility evaluation and treatment were ineligible. Patients who had received some form of infertility evaluation or treatment greater than 12 months ago were eligible.

### Data collection

Women were recruited from February 2013 to February 2014. At all study sites, bilingual (Spanish-English) research coordinators identified new patients from the complete list of patients being seen at each clinic. Telephone-based certified medical interpreters were utilized to recruit, consent, and complete study forms for patients speaking other languages. Coordinators identified new patients, presented the study, obtained written informed consent, and ensured study forms were completed. Study forms included a Fertility Knowledge Assessment and Social-Medical Demographics form (See Additional file 1). The coordinators then entered all data into a central de-identified database.

At UCSF’s HR clinic, due to impacted clinical schedule, an alternative recruitment strategy was utilized whereby a contact letter explaining the study was sent electronically to potential participants. Interested subjects were advised to complete the written consent form and the surveys and return them back by email or hard copy at their consultation. Forms that were returned without written consent were discarded. All data were then entered into the central de-identified database.

Fertility knowledge of participants was the primary outcome variable and was determined through a non-validated series of questions developed by the study team to query patients’ baseline fertility-related comprehension across multiple domains. Topics for items were identified through focus groups with infertility patients. Based on these topics, items were generated, reviewed by experts in the fields of reproductive health and urology, and tested in another small group of infertility patients. Problematic items were discarded. Data from responses for the quiz score was subjected to factor analysis to determine whether items corresponded with the overall score. The survey was produced in English and translated to Spanish.

The 11-question survey (Fertility Knowledge Assessment) included questions on basic infertility knowledge (anatomy and etiology of disease), prevalence, treatment, and questions pertaining to female and male infertility. Each response was recorded as a binary variable; correct answers were given a 1 point value and incorrect responses were given a 0 point value. The composite knowledge quiz score ranged 0–11 and percentage of questions correct was calculated for each subject. Internal consistency was measured with Cronbach’s alpha (alpha =0.69).

Demographic predictors including age, education, annual household income, region of origin, primary language, ethnicity, marital status, and occupation were determined through questionnaires. Medical demographics including parity, prior fertility treatment, number of ectopic pregnancies, therapeutic abortions, spontaneous abortions, and total prior pregnancies were additionally assessed. Education was dichotomized to Grade School/ High School, General Education Development diploma (GED) or Some College/Above***.*** 2010 US median household income census data was utilized to categorize subjects as follows: less than $25,000, $25,000–$50,000, $50,000–$75,000, $75,000–$100,000 and greater than $100,000. Country of origin was re-grouped to region of origin and included the United States, Mexico/South & Central America, Europe, Asia, and Other. Primary languages reported included English only, Spanish only, Mandarin, Cantonese, Vietnamese, Arabic, Tagalog, Korean, Bilingual, and Other. Based on response data, primary language was re-categorized to English, Spanish, and Other. Ethnicity was initially reported as White/European American/ Caucasian, African-American/Black, Latino/Hispanic, Asian, Pacific Islander, Native American, American Indian, Alaskan Native or Indigenous, Mixed/Multi-ethnic, and Do not know. Ethnicity was re-grouped to White, Hispanic, Black, Asian, or Other upon collection of responses. Partner status was dichotomized to Married or Unmarried (including single, never married, domestic partner) and occupation to Employed and Unemployed.

### Analysis

Descriptive statistics were utilized to describe the study population. Missing variables were not replaced. Bivariate comparisons were made between variables and quiz scores with Pearson’s chi-square analysis for categorical variables and linear regression for continuous variables. Linear regression was performed to investigate the association between baseline fertility knowledge and each outcome measure. Multivariate linear regression was used to examine relationships between baseline fertility knowledge and resource level while controlling for other predictor variables. Initial covariates were chosen a priori based on clinical relevance, literature review, and expert advice. Some variables were removed from the model to be parsimonious for *p* > 0.2. Constant variance was observed, residuals were normally distributed, and removal of outliers did not significantly affect regression coefficients or standard errors. Collinearity was investigated by assessing the variance inflation factor (VIF) for the model. All included variables had a VIF < 10 (mean 3.68). An Oaxaca Decomposition analysis was performed on the model but did not shed additional light on these findings beyond results presented with stratifying by resource level. Coefficients, 95% confidence intervals (CI), and *p* values were utilized to describe the results of regression models. Statistical significance was set at a level of *p < 0.2.* Stata 10 (Statacorp, College Station, TX, USA) was used for all analysis.

## Results

### Participant characteristics and infertility demographics across all study sites

A total of 143 women were included in the analysis: 49% participants were seen at LR clinics and 51% were seen at HR clinics. The mean age of women across all sites was 33.9 ± 5.7 years. Across all sites, the majority were partnered (67%,), born in the United States (54%), employed (77%), attended some college or above (71%), and had an annual household income of less than $25,000 (37%) (Table 1). Across all sites, the majority of patients had no prior infertility treatment (78%) and 55% of women had achieved prior pregnancy. The mean fertility knowledge score across all sites was 6.85 ± 2.5 points out of 11 points.

**Table 1:** Social & Medical Demographics among Infertility Cohort (*n* = 143)

	SFGH (LR)		Mt. Zion (HR)		Ben Taub (LR)		Texas children’s (HR)	
	(*n* = 43)		(*n* = 33)		(*n* = 27)		(*n* = 40)	
	*N*	%	*N*	%	*N*	%	*N*	%
**Region of origin**								
Native United States	12	27.9	20	60.6	6	22.2	27	67.5
Mexico/Central & South America	21	48.8	1	3.0	15	55.6	4	10.0
Europe	2	4.7	3	9.1	0	0.0	2	5.0
Asia	7	16.3	7	21.2	2	7.4	6	15.0
Other	1	2.3	2	6.1	4	14.8	1	2.5
**Ethnicity**								
White	3	7.0	17	51.5	2	7.4	13	32.5
Hispanic	22	51.2	1	3.0	16	59.3	7	17.5
Black	6	14.0	4	12.1	8	29.6	9	22.5
Asian	8	18.6	7	21.2	1	3.7	8	20.0
Other	4	9.3	4	12.1	0	0.0	3	7.5
**Education**								
High School or less	18	41.9	2	6.1	19	70.4	2	5.0
Some College or Above	25	58.1	31	93.9	8	29.6	38	95.0
**Primary language**								
English	12	27.9	24	72.7	7	25.9	26	65.0
Spanish	17	39.5	0	0.0	13	48.2	1	2.5
Other	14	32.6	9	27.3	7	26.0	13	32.5
**Marital status**								
Married	21	48.8	26	78.8	18	66.7	31	77.5
Unmarried	22	51.2	7	21.2	9	33.3	9	22.5
**Occupation**								
Employed	27	62.8	33	100.0	14	51.9	37	92.5
Unemployed	16	37.2	0	0.0	13	48.1	3	7.5
**Household income ($)**								
< 25,000	27	62.8	1	3.0	23	85.2	2	5.0
25,000-50,000	10	23.3	0	0.0	4	14.8	3	7.5
50,000-75,000	1	2.3	3	9.1	0	0.0	9	22.5
75,000-100,000	1	2.3	1	3.0	0	0.0	9	22.5
> 100,000	0	0.0	28	84.9	0	0.0	16	40.0
Did not wish to report	4	9.3	0	0.0	0	0	1	2.5
**Parity**								
Yes	22	51.2	19	57.6	16	59.3	22	55.0
No	21	48.8	14	42.4	11	40.7	18	45.0
**Prior treatment**								
Yes	4	9.3	16	48.5	0	0.0	1	2.5
No	29	67.4	17	51.5	27	100.0	39	97.5
Did not wish to report	10	23.3	0	0.0	0	0.0	0	0.0

### Participant characteristics and infertility demographics: HR versus LR settings

As mentioned above, 70 (49%) women were from LR sites while 73 (51%) were from HR sites (Table 1). The mean age of women from LR clinics was 32.8 ± 6.1 years. The majority were from Mexico/Central/South America (51%), were partnered (55%), of Hispanic descent (54%), completed grade or high school (52%), employed (58%), spoke Spanish (42%) followed by Other (non-English languages) (30%), had an annual income less than $25,000 (71%), and had not received any form of prior infertility treatment (80%). Of note, 54% of women within this group reported prior pregnancy. The mean Fertility Knowledge Assessment quiz score within the low-resource population was 5.3 ± 2.3 points out of 11.

Comparatively, the mean age of the HR group was 35.0 ± 5.0 years. The majority were from the United States (non-immigrant) (64%), partnered (78%), White (41%), completed some college or above (95%), employed (96%), spoke English (68%), had an annual household income greater than $100,000 (60%), and had not received any form of fertility treatment (76%). In this group, 57% reported prior pregnancy. The mean fertility knowledge assessment score in this group was 8.04 ± 2.3 points out of 11.

A one-way ANOVA demonstrated a statistically significant difference in fertility knowledge scores between patients from LR and HR settings. Further analysis (Table 2) demonstrated that on average, women from HR settings scored 3.0 points higher on the fertility knowledge assessment, more than a full standard deviation greater than their LR counterparts. Within the LR group, individuals from Mexico/Central and South America scored 3.0 points less compared to individuals from the United States LR group. Women of Hispanic ethnicity had a 3.4-point lower quiz score compared to white women in the same group. In addition, women who reported some college or above scored 2.97 points higher than those who only completed grade school/ high school. Individuals with a household income of $50–75,000 achieved 3.1 points higher than those women earning less than $25,000 while those who earned between $75–100,000 achieved 3.3 points compared to the reference group (<$25,000 household income).

**Table 2:** Bivariate relationships between socio-demographic and fertility knowledge score

	β	95% CI	*p* value
**Age**	0.04	−0.03 - 0.11	0.28
**Location**			
SFGH (Low-resource)	Ref	Ref	Ref
Mt. Zion (High-resource)	3.09	2.15–4.04	< 0.001
BT (Low-resource)	0.30	−0.69 - 1.31	0.54
TCH (High-resource)	3.11	2.22–4.01	< 0.001
**Resource Level**			
Low Resource	Ref	Ref	Ref
High Resource	2.99	2.31–3.66	< 0.001
**Region of Origin**			
Native United States	Ref	Ref	Ref
Mexico/Central & South America	−3.00	−3.86 - -2.15	< 0.001
Europe	0.95	−0.76 - 2.66	0.27
Asia	−0.95	−2.01 - 0.11	0.08
Other	−1.66	−3.27 - 0.5	0.04
**Ethnicity**			
White	Ref	Ref	Ref
Hispanic	−3.41	−4.37 - -2.45	< 0.001
Black	−1.42	−2.52 - -0.32	0.01
Asian	− 1.30	−2.42 - -0.16	0.03
Other	−0.28	− 1.75 - 1.20	0.71
**Annual household Income**			
< $25,000	Ref	Ref	Ref
$25,000-50,000	1.07	−0.06 - 2.20	0.06
$50,000-75,000	3.10	1.85–4.36	< 0.001
$75,000-100,000	3.35	2.00–4.70	< 0.001
> $100,000	3.06	2.23–3.86	< 0.001
Did not wish to report	−1.08	−2.98 - 0.82	0.26
**Education**			
High School or less	Ref	Ref	Ref
Some College or Above	2.97	2.19–3.76	< 0.001
**Parity**			
No	Ref	Ref	Ref
Yes	−0.58	−1.42 - 0.26	0.18
**Prior fertility treatment**			
No	Ref	Ref	Ref
Yes	0.96	−0.20 - 2.12	0.10
Did not wish to report	−1.95	−3.56- -0.38	0.02
**Primary Language**			
English	Ref	Ref	Ref
Spanish	−3.24	−4.30- -2.30	< 0.001
Other	−1.09	−1.94- -0.24	0.01
**Marital Status**			
Married	Ref	Ref	Ref
Unmarried	−0.73	−1.62- 1.53	0.11
**Occupation**			
Employed	Ref	Ref	Ref
Unemployed	−2.75	−3.65- -1.85	< 0.001

Upon multivariate analysis (Table 3), the relationships between resource-level, age, region of origin, prior treatment, prior pregnancy, language and marital status disappeared. The sole remaining independent factor associated with fertility scores was education-level. Analyzed separately, in the LR group, household income level (*p* > 0.2 for all levels) and educational level (*p* = 0.16) was not related to knowledge score. In the HR setting, household income was strongly associated with quiz score results (*p* < 0.02 for all levels of income greater than $25,000 per year) after holding all other variables constant including education.

**Table 3:** Multivariate relationships between socio-demographic and fertility knowledge score

	β	95% CI	*p* value
**Resource level**			
Low-Resource	Ref	Ref	Ref
High-Resource	0.84	−0.61 - 2.28	0.25
**Region of origin**			
Native United States	Ref	Ref	Ref
Mexico/Central & South America	0.09	−1.98 - 2.18	0.93
Europe	0.24	− 1.63 - 2.12	0.80
Asia	−1.40	−3.63 - 0.82	0.21
Other	−0.26	−2.00 - 1.49	0.77
**Ethnicity**			
White	Ref	Ref	Ref
Hispanic	−1.54	−3.28 - 0.20	0.08
Black	−0.51	−1.76 - 0.73	0.41
Asian	0.49	−1.51 - 2.49	0.63
Other	0.35	−1.04 - 1.73	0.62
**Annual household income**			
< $25,000	Ref	Ref	Ref
$25,000-50,000	0.49	−0.66 - 1.64	0.40
$50,000-75,000	1.23	−0.60 - 3.06	0.19
$75,000-100,000	1.50	−0.39 - 3.40	0.12
> $100,000	0.84	−0.83 - 2.50	0.32
Did not wish to report	−0.94	−2.80 - 0.92	0.32
**Education**			
High School or Less	Ref	Ref	Ref
Some College or Above	0.95	0.003–1.90	0.05
**Primary language**			
English	Ref	Ref	Ref
Spanish	0.22	−1.65 - 2.08	0.82
Other	0.17	−0.98 - 1.32	0.77
**Occupation**			
Employed	Ref	Ref	Ref
Unemployed	−0.85	−1.77 - 0.070	0.07

## Discussion

Involuntary childlessness often becomes a central and preoccupying issue in infertile patients’ lives; this can be compounded by lack of access to basic care and fertility treatment. Direct barriers to fertility care include cost of treatment, marital status, employment, and insurance type [20–22]. These barriers are often magnified in immigrant communities, a growing but highly marginalized demographic in the United States that has been shown in many domains of women’s health to have adverse health outcomes due to delays in, and limited, access to care [23, 24].

Our study is the first comparative evaluation of health literacy and influencing factors among women across different sociocultural and income backgrounds presenting for fertility care in metropolitan centers in the United States. The principal strength of our study is our ability to access socio-culturally, largely immigrant women seeking infertility care, a population that is challenging to access as there are relatively few public health centers that provide fertility services by specialized providers to this population. We recruited subjects in-person through infertility clinics based at public hospitals and thus were able to access populations that may have limited access to Internet and/or social media sites.

Pioneering studies by Becker and Nachtigall conducted at San Francisco General Hospital (SFGH) showed that difficulty finding a physician, legal immigration status, and language limitations provide additional barriers in accessing fertility care [25, 26]. Moreover, they concluded that the combination of communication, comprehension, continuity, bureaucracy, accessibility, and affordability presented significant challenges in obtaining infertility care to many low-income immigrant Latino patients [25, 26]. Similar barriers to care were identified in a study of clinical vignettes of immigrant women presenting for care to a public hospital in Boston, Massachusetts [27]. Additionally, Ho et al. in a study of patients at SFGH found that level of education and socioeconomic status was inversely proportionate to duration of infertility [28]. Recently, Wiltshire et al. assessed infertility knowledge and treatment beliefs among African American women from an urban community in Atlanta, Georgia using a field-tested survey from the International Fertility Decision Making Study [29]. In this population, a lower level of infertility knowledge was identified and, similar to our findings, higher level of education correlated with increased knowledge scores [29].

The data from prior studies of populations with higher socioeconomic status and health literacy identified several factors strongly associated with fertility knowledge including resource level, region of origin, ethnicity, primary language, education level, and parity [30, 31]. These individual and interrelated characteristics are particularly relevant to understand as each present a barrier in access to fertility care. Our data demonstrate that education level remained independently associated with knowledge after adjustment for all factors in our model. This finding aligns with studies in other domains of reproductive health that indicate health literacy correlates with increased education level [32–34]. In our study, however, after analyzing LR and HR groups separately, education was no longer a significant factor associated with fertility knowledge in each group, suggesting that identifying those with attainment of formal academic education was insufficient. Reproductive knowledge may not be something learned in the formal education process but acquired from other sources that are resource setting dependent. In fact, among the HR participants, having an annual household income more than $25,000 was strongly associated with higher fertility knowledge but not attainment of higher educational levels. It is challenging to discern “true” relationships as both income and education are highly related to resource setting.

Identification and recognition of factors associated with lower health literacy are critical to develop targeted interventional strategies to improve fertility knowledge. For example, clinicians could develop additional Spanish language written or video resources to educate their patients about reproductive options as part of their initial visit, thereby focusing on primary language as a barrier to reproductive knowledge. Recently, Garcia et al. found that regardless of education level, tailored oral education significantly increased fertility knowledge in a group of oocyte donors [14]. Moreover, Anspach et al. surveyed medical students and house staff on their professional and personal perception of fertility before and after an educational intervention. They found that both groups increased their score post-intervention in the knowledge-based questions [35]. While this study assessed a distinct population of medical students and staff, it demonstrates the value of education to promote fertility-related knowledge. In this stride, our findings will potentially help direct resources toward improved education via seminars/educational materials to increase awareness and, ultimately, earlier presentation to care.

There are several limitations of this study. In the absence of a well-validated fertility knowledge instrument available at the inception of our study, we developed a tool to assess fertility knowledge. Of note, in 2017 Kudesia et al. developed the Fertility and Infertility Treatment Knowledge Score (FIT-KS), which is a short survey, utilized as a quick assessment of fertility knowledge [16]. Although our tool was trialed on a small group of research coordinators and providers, formal validation of the instrument has not been undertaken. Additionally, recall bias was a limitation of the study as we utilized self-report instruments.

Furthermore, given the option for UCSF HR participants to mail or email completed study forms, this could have contributed to a selection bias to favor those who have access to computers; however, since in HR clinical settings, e-communication is one of the main routes of nurse-patient communication, we suggest that this bias was unlikely to contribute to skewed results. Future studies should examine sources of participants’ fertility knowledge and how prior pregnancy and infertility treatment impacts fertility health literacy. Fertility knowledge is essential for patients to access care and appropriate therapeutics. In no other population setting is this need likely greater than in the growing and diverse immigrant populations of the United States. Designing and implementing a fertility education program and tools for patients from these communities and assessing the impact of such educational efforts is an important next step.

## Conclusions

Women from low-resource, largely immigrant communities, seeking fertility care have greater disparities in fertility knowledge and lower health literacy. Further studies are needed to understand these barriers and to develop targeted inventions to lower disparities and improve care for these vulnerable populations.

## Supplementary information

**Additional file 1.**

## Data Availability

The data that support the findings of this study are available from the corresponding author upon reasonable request.

## Abbreviations

SD: Standard deviation
CI: Confidence interval
UCSF: University of California, San Francisco
BCM: Baylor College of Medicine
HR: High-resource
LR: Low-resource
IUI: Intra-uterine insemination
IVF: In vitro fertilization
ART: Assisted reproductive technology
IFDMS: International Fertility Decision-making Study
FIT-KS: Fertility & Infertility Treatment Knowledge Score
