# Supplementary material for: Disparities in fertility knowledge among women from low and high resource settings presenting for fertility care in two United States metropolitan centers
Source: Fertil Res Pract. 2020 Aug 15;6:15. doi: 10.1186/s40738-020-00084-1 (PMC7429753; doi:10.1186/s40738-020-00084-1)
Supplement: Supplementary file 1 — Additional file 1. [file 40738_2020_84_MOESM1_ESM.docx]

**Fertility Knowledge Assessment**

(1) In what part of the body does the sperm normally fertilize (combine with) the egg?

- - Ovary
  - Fallopian Tube
  - Uterus (womb)
  - Cervix

(2) How many eggs does a fertile woman normally release (ovulate) each month?

- - 1
  - 3
  - 5
  - 10

(3) Sexually transmitted infections (infections passed through sexual intercourse) can lead to infertility in some women.

- - True - sexually transmitted infections, particularly when untreated, can lead to scarring of

the fallopian tubes and are a common cause of infertility worldwide.

- - False - sexually transmitted infections rarely impact the fertile potential of a woman.

(4) Men contribute to an estimated 30 to 50% cases of infertility among couples.

- - True - Men are an important cause of infertility in couples.
  - False - Men contribute to only a small fraction of infertile cases.

(5) Most women can easily get pregnant when they are 40 years of age.

- - True - Women can easily get pregnant at age 40 when they want to have a baby.
  - False - it is naturally very difficult for most women to conceive past age 40, even with use of strong fertility treatments.

(6) In vitro fertilization (IVF) refers to an infertility treatment in which:

- - A man’s sperm is placed past the cervix directly inside the uterus.
  - A man’s sperm and woman’s egg are combined inside a laboratory and the resulting embryo is transferred into the uterus (womb).
  - When a woman carries a pregnancy for another couple that cannot get pregnant.
  - Surgery is performed to harvest sperm from the man

(7) If a woman wants to get pregnant, when is the best time to have sexual intercourse in order to conceive (the optimal fertile window):

- - First three days after her period
  - Midway in her cycle
  - Three days prior to onset of a woman’s period

(8) Having an abortion lowers the chance of pregnancy later in life.

- - True – An abortion when performed in safe, medical circumstances can make it harder to get pregnant later in life.
  - False- An abortion when performed in safe, medical circumstances does not affect the chance of a woman to get pregnant in later life.

(9) The cost of a single in vitro fertilization cycle in the United States is

approximately:

- - $1,000
  - $12,000
  - $20,000
  - $100,000

(10) Stress in women is an important cause of infertility.

- - True - Stress is an important cause of infertility among women
  - False - Infertility can lead to stress but stress itself is not considered usually a primary cause of infertility.

(11) At a woman’s peak fertility, her chance of getting pregnant naturally through intercourse per month is:

- - 10%
  - 30%
  - 50%
  - 70%

**Social Demographics Survey**

(1) What is your gender?

- - Male
  - Female

(2) In what year were you born? _____________

(3) In what country were you born? ________________

(4) How long have you been in the United States? __________

(5) What do you consider your ethnicity to be? (Check all that apply)

- - White/European American/ Caucasian
  - African-American/Black
  - Latino/Hispanic
  - Asian
  - Pacific Islander
  - Native American, American Indian, Alaskan Native or Indigenous
  - Mixed/Multi-ethnic: Please specify if possible: ______________
  - Do not know

(6) What is the primary language spoken at home?

- - English only
  - Spanish Only
  - Mandarin
  - Cantonese
  - Vietnamese
  - Arabic
  - Tagalog
  - Korean
  - Bilingual: Which languages?______________
  - Other: _______________

(7) What is your religion?

- - Protestantism
  - Catholicism
  - Christianity
  - Judaism
  - Atheism/agnosticism
  - Buddhism
  - Islam
  - Other:_________________

(8) What is your marital status?

- - Married
  - Unmarried (single, never married, domestic partner)

(9) Number of total years married? ________

(10) Number of previous marriages (not including current marriage)? ________

(11) Number of children from previous marriages? ______

(12) Have you been pregnant before?

- - Yes
  - No

(13) If yes, how many times? _____

(14) How many children do you have? ______

If you have children:

Age of children: _____

Gender of Children:

- - Male
  - Female
  - Other_________

(15) What is the highest degree or level of school you have completed?

- - Grade school
  - High School, GED
  - Some college
  - College degree
  - Graduate degree

(16) What is your current or previous Occupation?

- - Administrative/Technical
  - Clerical/Sales
  - Skilled Labor
  - Unskilled Labor
  - Homemaker

(17) What is your total household income?

- - Less than $25,000
  - $25,000-$50,000
  - $50,000-$75,000
  - $75,000-$100,000
  - $100,000-$150,000
  - $150,000-$200,000
  - $200,000-$250,000
  - More than $250,0000

(18) What best describes your medical insurance?

- - Private Health Insurance
  - Public Health Assistance Program (including, for example, Healthy San Francisco)
  - No Health Insurance
  - Other

(19) Does your insurance cover fertility care for either diagnosis or treatment?

- - Yes
  - No
  - I am not sure

(20) Have you previously sought any form of infertility treatment?

- - Yes
  - No
  - I am not sure

(21) What type of infertility treatment have you previously had?

- - Medications
  - Medications with intrauterine insemination
  - In Vitro Fertilization (IVF)
  - Other form of treatment
  - No treatment

(22) If other form of treatment selected above, what type of treatment:_____________________

(20) Please indicate if you have had any of the following (check all that apply).

Ectopic pregnancy ______________times

Therapeutic Abortion _____________times

Miscarriage (spontaneous abortions)_____________times

Stillbirth ____________times
